# Effect of micro/nano-sheet array structures on the osteo-immunomodulation of macrophages

**DOI:** 10.1093/rb/rbac075

**Published:** 2022-10-04

**Authors:** Xinhui Zheng, Lan Chen, Ji Tan, Jianhua Miao, Xuanyong Liu, Tieyi Yang, Zhihong Ding

**Affiliations:** Department of Orthopedics, Gongli Hospital of Shanghai Pudong New Area, Shanghai 200135, China; Department of Orthopedics, Zhengzhou Central Hospital Affiliated Zhengzhou University, Zhengzhou 450007, China; State Key Laboratory of High-Performance Ceramics and Superfine Microstructure, Shanghai Institute of Ceramics, Chinese Academy of Sciences, Shanghai 200050, China; School of Materials Science and Engineering & Henan Key Laboratory of Advanced Magnesium Alloy & Key Laboratory of Materials Processing and Mold Technology (Ministry of Education), Zhengzhou University, Zhengzhou 450001, China; State Key Laboratory of High-Performance Ceramics and Superfine Microstructure, Shanghai Institute of Ceramics, Chinese Academy of Sciences, Shanghai 200050, China; Department of Orthopedics, Zhengzhou Central Hospital Affiliated Zhengzhou University, Zhengzhou 450007, China; State Key Laboratory of High-Performance Ceramics and Superfine Microstructure, Shanghai Institute of Ceramics, Chinese Academy of Sciences, Shanghai 200050, China; Department of Orthopedics, Gongli Hospital of Shanghai Pudong New Area, Shanghai 200135, China; Department of Orthopedics, Gongli Hospital of Shanghai Pudong New Area, Shanghai 200135, China

**Keywords:** micro-nano topography, immunomodulation, macrophages, osteogenesis

## Abstract

The immune response induced by surface topography crucially determines the implant success. However, how the immune response is mediated by the size of surface topography remains unclear. Hence, various biocompatible Mg-Al layered double hydroxides sheet-array films with different sizes (nano, micro and nano/micro mixture) were constructed on the biomedical titanium, and their osteo-immunomodulation effects on the macrophages were explored. The nano-sheet array structures significantly promoted the polarization of M2 macrophages by activating the PI3K-AKT-mTOR signaling pathway with high gene expressions of integrin β2 and FAK. While the micro-sheet array structures enhanced osteogenic differentiation of mouse bone marrow mesenchymal stem cells (mBMSCs) *via* ROCK-YAP/TAZ-mediated mechanotransduction. Moreover, the nano-sheet array structures promoted the osteogenic differentiation of mBMSCs with a high proportion of M2 macrophages through a shared medium. This study gave further information concerning integrin-induced focal adhesions in cells of different sheet array structures and their role in macrophage polarization and osteogenic differentiation of mBMSCs, which might help to design biomaterial surfaces with optimal geometry for a desired immunemodulation.

## Graphical Abstract

**Graphical Abstract:**
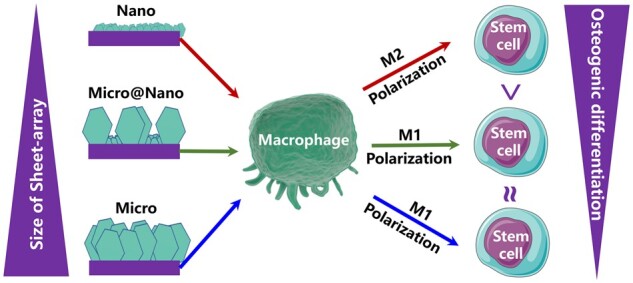

## Introduction

The surface morphology of biomaterials is closely related to the cell adhesion behavior, the formation of adhesive spots and the relevant signaling transmission, which is extremely important for orthopedic implants to obtain osseointegration [1, 2]. One example is extracellular matrix (ECM), a complex network of nanopores and nanofibers. In the past few decades, quite a number of researchers have devoted themselves to endowing implants with enhanced osseointegration by constructing specific micro/nano surface topography [3, 4]. Dalby’s work revealed that the completely ordered or completely random nanopits could maintain multipotency of human mesenchymal stem cells (hMSCs) [5], while the slightly irregular substrates did promote hMSCs to differentiate toward osteogenic lineages directly [6]. Pan *et al*. [7] fabricated a hierarchical macropore/nanowire surface to improve the osteogenic performance of osteoblast cell lines through the development of cytoskeleton and ROCK-regulated cytoskeleton tension. However, the cascade of clinical researches has shown that the results of *in vitro* and *in vivo* experiments sometimes are inconsistent. This is mainly because the internal environment is far more complex than the *in vitro* simulation environment, and the important role of immune response in bone integration was always ignored in previous *in vitro* studies. Osteoimmunology studies have demonstrated that the immune system and skeletal system are closely related, sharing many cytokines, signaling molecules, receptors and transcription factors [8, 9]. An overactive and long-term inflammatory reaction often results in a fibrous envelope formed around the biomaterials, which is not conducive to the binding of implants and the bone tissue [10, 11]. An ideal implant biomaterial should promote the rapid resolution of inflammation and a successful osseointegration process. Therefore, the immune response induced by biomaterials is one of the key factors that determine the fate of biomaterials implanted *in vivo*, and the researchers on biomaterials need to consider the immune response caused by the biomaterials comprehensively.

Macrophage, as the first line of defense of host immune response, plays an important role in the immune response induced by biomaterials and bone repair response [12]. Macrophages can polarize into two main phenotypes, the classically activated M1 and the alternative active M2, which are highly dynamic and plastic in response to the stimuli. The M1 phenotypes are commonly considered as the pro-inflammatory macrophages and secrete plenty of pro-inflammatory cytokines, including tumor necrosis factor-α (TNF-α) and interleukin-6 (IL-6) to kill bacteria and other pathogens [13]. Whereas the M2 phenotypes produce a massive number of anti-inflammatory cytokines, e.g. interleukin-1 receptor a and interleukin-10 (IL-10) to promote inflammation resolution [14]. They are also able to promote new bone formation as well as ECM reconstruction through secreting various growth factors, such as vascular endothelial growth factor and transforming growth factor-β. The change of macrophages from the M1 phenotype to the M2 phenotype is a symbol of the transformation of the microenvironment around the implant from inflammatory response and catabolism to bone tissue regeneration and anabolism [15]. The desirable biomaterials should be able to regulate the proportion of M1/M2 macrophage to obtain satisfactory immunomodulatory properties and build an osteogenesis-enhancing microenvironment.

Research continues to validate that the physical properties of implant surface, including certain micro/nano-structure and surface wettability can induce macrophages polarization, thus influencing the subsequent osteogenesis possess [16, 17]. He *et al*. [18] demonstrated that the Ti implant surface coated with about 100-nm diameter titanic nanotubes (NT-100) can promote M1 macrophages polarization, which was related to FAK-MAPKs signaling, particularly the JNK-ERK1/2 signaling pathway. Our previous study also confirmed that nanostructures with different surface elastic moduli regulate immune responses of macrophages *via* the FAK-NF-κB signaling pathway [19]. However, how the polarization of the macrophages is regulated by the size of the surface topography remains unclear, which has greatly limited the development of implant surface design. Layered double hydroxides (LDHs) are a class of lamellar materials and made up of positively charged brucite-like layers and an interlayer containing various charge-balanced anions, which have good biocompatibility [20, 21]. LDHs film that has a regular micro/nano-sheet array structure can be easily fabricated on the metal implant surface by hydrothermal treatment. Besides, the element composition and the size of LDHs can be regulated by adjusting hydrothermal conditions. Therefore, LDHs film is an appropriate model to study the effect of surface morphology on the immune response of macrophages and later osteogenic properties.

In this study, the biocompatible Mg-Al LDHs films with micro, nano and micro/nano multilevel sheet array topography were constructed on the biomedical titanium through a simple hydrothermal treatment. The detailed interactions between the micro/nano-sheet and macrophages, and the regulation of the immune microenvironment on osteogenesis were investigated. These studies were to provide a full understanding of the immunomodulatory properties of a desirable implant surface and give a short glimpse of its immune response *in vivo*.

## Materials and methods

### Sample preparation

Commercially titanium plates were machined into different dimensions. The samples in the sizes of 20 × 20 × 1 mm were used in the flow cytometry and the real-time polymerase chain reaction (RT-PCR) tests. The other test samples were in the dimensions of 10 × 10 × 1 mm. All the samples were firstly ultrasonically cleaned with a mixed acid solution (HNO_3_:HF:H_2_O = 5:1:4) three times, 5 min each time. Next, the samples were ultrasonically cleaned with distilled water twice for 5 min each time and then dried for use. The pretreated sample was named the Ti sample. Then, Mg-Al LDHs films were constructed on the Ti surface by hydrothermal treatment with a mixed reaction solution consisting of Mg(NO_3_)_2_, Al(NO_3_)_3_ and urea. The reaction conditions were shown in Supplementary Table S1 [22]. Finally, the samples were rinsed with ultrapure water and dried at room temperature.

### Surface structure and chemical characterization

Scanning electron microscopy (SEM, S-4800, Hitachi, Japan) was used to observe the surface morphologies of the samples. X-ray diffraction (XRD, D/Max, Rigaku, Japan) patterns were acquired with a Cu Kα radiation (λ = 1.5411 Å). X-ray photoelectron spectroscopy (XPS, PHI-5000C ESCA System PerkinElmer, USA) was used to detect the chemical compositions and chemical states of the samples.

### *In vitro* studies

#### Immunological evaluation

##### Macrophage culture

Mouse mononuclear-macrophage leukemia cell line (RAW264.7; cells were kindly provided by Cell Bank, Chinese Academy of Sciences, Shanghai, China) was used to evaluate the immune response of the samples *in vitro*. Macrophages were cultured in a humidified atmosphere of 5% CO_2_ at 37°C. The complete cell culture medium consisted of 84% high glucose DMEM medium (Gibco, USA), 15% FBS (Gibco, USA) and 1% penicillin/streptomycin (Antibiotic/Antimycotic; Gibco, USA). Cells were passaged at a ratio of 1:3 every 3 days. All the samples were sterilized with 75% ethanol for 2 h before cell experiments [19].

##### Cell proliferation and morphology

The cell proliferation and viability were measured by using the alamarBlue™ (Thermo Fisher Scientific Inc., USA) assay. First, 1 × 10^5^ cells per well were seeded on the sample surfaces (three replicates) on 24-well plates for 4 h, 1 day and 4 days. For each incubation time, the samples were rinsed with PBS. Next, 0.5 ml fresh medium with 10% alamarBlue™ was added and cultured for 2 h. Then, 0.1 ml medium was added into a black 96-well plate to detect the fluorescence intensity (the wavelength of excitation/emission = 560/590 nm).

Cells were fixed with 0.5 ml 2.5% glutaraldehyde cultured for 1 day in the dark. Then, cells were dehydrated by a series of ethanol solutions (30, 50, 75, 90 and 100 v%) and dried by a series of hexamethyl disilylamine/ethanol solutions (v/v = 1:2, 1:1, 2:1 and 1:0). The cell morphologies were observed with the SEM (S-3400, Hitachi, Japan) at 5 kV accelerated voltage.

##### Immunofluorescence staining

Macrophages with a density of 1 × 10^5^ cells per well were seeded on sample surfaces and cultured for 1 day. Cells were fixed with paraformaldehyde (PFA; 4%) for 18 h at 4°C, then rinsed with PBS three times. Cells were permeabilized with 0.1% (v/v) Triton X-100 (Amresco, USA) for 2 min, and blocked Fc-receptor with 1 wt% BSA (Sigma-Aldrich, USA) for 30 min. Afterward, cells were incubated with the primary antibodies anti-CD206 (1:50; Abcam, UK) and against-iNOS (1:50; Novus, USA) for 12 h at 4°C in the dark. Then, cells were incubated with donkey anti-mouse IgG H&L Alexa Fluor 594 (1:200; Abcam, UK) and donkey anti-rabbit IgG H&L Alexa Fluor 488 (1:200; Thermo Fisher Scientific Inc., USA) secondary antibodies for 2 h in the dark. Finally, cellular nuclei were stained with 4′,6′-diamidino-2-phenylindole (1:1000; Thermo Fisher Scientific Inc., USA) for 10 min at room temperature in the dark. The confocal laser scanning microscope (Leica SP8, Germany) was used to observe the staining images.

##### Flow cytometry

Macrophages with a density of 6 × 10^5^ cells per well were seeded on the samples in 6-well plates and cultured for 4 days. Cells were collected, centrifuged at 300 g for 5 min at 4°C. Cells were resuspended with PBS solution and the concentration of cells to be detected was adjusted to 10^6^ cell/ml. Next, cells were incubated with the purified rat anti-mouse CD16/CD32 antibody (BD Pharmingen, USA) to block Fc-receptors for 10 min at room temperature. The detected cells were incubated with phycoerythrin-conjugated anti-mouse F4/80 antibody (Thermo Fisher Scientific Inc., USA) to mark macrophages. Then, cells were incubated with fluorescein isothiocyanate (FITC)-conjugated anti-mouse CCR7 antibody (Bioss, China) or FITC-conjugated anti-mouse CD206 antibody (Thermo Fisher Scientific Inc., USA) on ice for 30 min in the dark to mark M1 phenotype and M2 phenotype, respectively. Cells were rinsed twice with PBS and transferred to FACS tubes (0.5 ml per tube) for test using flow cytometer (CytoFLEX, Beckman, USA), 10 000 events per tube were analyzed by using FlowJo™ v10 software.

##### Enzyme-linked immunosorbent assay

Macrophages with a density of 1 × 10^5^ cells per well were seeded on the samples (three replicates) for 4 days. The cell culture medium was collected, centrifuged at 1500 rpm at 4°C to get the supernatant, and stored in the sterile 1.5 ml tube for use. The supernatant of culture medium was used to measure the concentration of interleukin-4 (IL-4; Anogen, Canada), IL-6 (Anogen, Canada), IL-10 (Raybiotech, USA) and TNF-α (Anogen, Canada) by enzyme-linked immunosorbent assay (ELISA). The absorbance of the plate was detected by a microplate reader according to the protocol. The concentrations of the cytokines were calculated by using the corresponding standard curves.

##### RT-PCR analysis

Cells were cultured for 4 days on samples with an initial density of 5 × 10^5^ cells per well, and the total RNA was extracted using TRIzol™ reagent (Thermo Fisher Scientific Inc., USA). Complementary DNA (cDNA) was synthesized from the extracted RNA using Transcriptor First Strand cDNA Synthesis Kit (Roche, Switzerland). RT-PCR test was conducted on the LightCycler^®^ 480 system (Roche, Switzerland) using LightCycler^®^ 480 SYBR Green I Master (Roche, Switzerland). GAPDH was selected as the reference gene, the used primers were listed in Supplementary Table S2, and they were purchased from BioTNT. The target gene expression levels relative to the reference gene were calculated by 2^−ΔΔCt^ analysis method and quantified using the comparative threshold method. RT-PCR experiments were performed at least twice each, and each sample was analyzed in triplicate.

#### Induction of osteogenic differentiation by macrophages

##### Mouse bone marrow mesenchymal stem cells culture

Mouse bone marrow mesenchymal stem cells (mBMSCs; cells were kindly provided by Sciencell Biotechnology Co., Ltd, USA) were used to study the induction of macrophage polarization on osteogenic differentiation of stem cells. The mBMSCs were cultured in a humidified atmosphere of 5% CO_2_ at 37°C. The complete culture medium for mBMSCs was the same as that for macrophages. Cells were passaged at a ratio of 1:3 every 3 days, and the primary mBMSCs used in the experiments were passaged within five times. All the samples were also sterilized with 75% ethanol for 2 h before cell experiments.

##### Establishment of indirect co-culture model

The conditioned medium of macrophages cultured on samples was collected as described in section ‘Enzyme-linked immunosorbent assay’. The cell culture medium of macrophages cultured on different samples (Ti, Micro, NW and Nano) for 4 days was collected separately and centrifuged to obtain supernatant, which was called macrophage-conditioned medium. Then, the macrophage-conditioned medium was mixed with the fresh DMEM complete medium at 1:1 to culture mBMSCs and study the effect of macrophages on osteogenic differentiation of mBMSCs. The mBMSCs were cultured on the four groups of samples using the mixed macrophage-conditioned medium collected from the corresponding sample surface. A schematic diagram of the indirect co-culture model was shown in Supplementary Fig. S1.

##### ALP activity assay

Stem cells with an initial density of 0.5 × 10^4^ cells per well were seeded on the samples and cultured for 10 days to evaluate the ALP activity. For the ALP staining test, cells were rinsed with PBS twice and fixed with 4% PFA for 10 min. Then, cells were incubated with BCIP/NBT working solutions (Beyotime-Biotech Co., China) for 2 h in the dark at room temperature and rinsed with ultrapure water. Stained cells were visualized by a fluorescence microscope (Olympus IX71, Japan). For quantity analysis, cells on samples (four replicates) were cracked for 40 min by using lysis buffer on ice, and centrifuged at 8000 rpm for 10 min at 4°C and collected. Next, cells were incubated with p-nitrophenyl phosphate for 30 min at 37°C. Then, NaOH solution (1 M) was added to terminate the reaction. The total ALP activity was measured by detecting the absorbance at 405 nm wavelength. The total intracellular protein levels were quantified by detecting absorbance at 562 nm using bicinchoninic acid (BCA) kit (Thermo Fisher Scientific Inc., USA). Finally, the relative ALP activities were normalized to the total protein and were presented as mM/mg total proteins.

##### RT-PCR analysis

RT-PCR test was used to measure the expression of osteogenic genes (BMP-2, OPN and OCN) in mBMSCs and macrophage-induced mBMSCs to evaluate the osteogenic differentiation. The detailed process was as described in the previous section ‘RT-PCR analysis’. The primers used in the RT-PCR test were listed in Supplementary Table S3 and they were also purchased from BioTNT.

### Statistical analysis

GraphPad Prism software was used for statistical analysis of data, and the results of each group were expressed as mean ± standard deviation. Statistically significant differences (*P*) were analyzed by one-way analysis of variance and SNK-q tests between groups. A value of *P* < 0.05 was considered statically significant and was represented by the symbol ‘*’; *P* < 0.01 was ‘**’; *P* < 0.001 was ‘***’.

## Results

### Surface characterization

**Figure 1.**
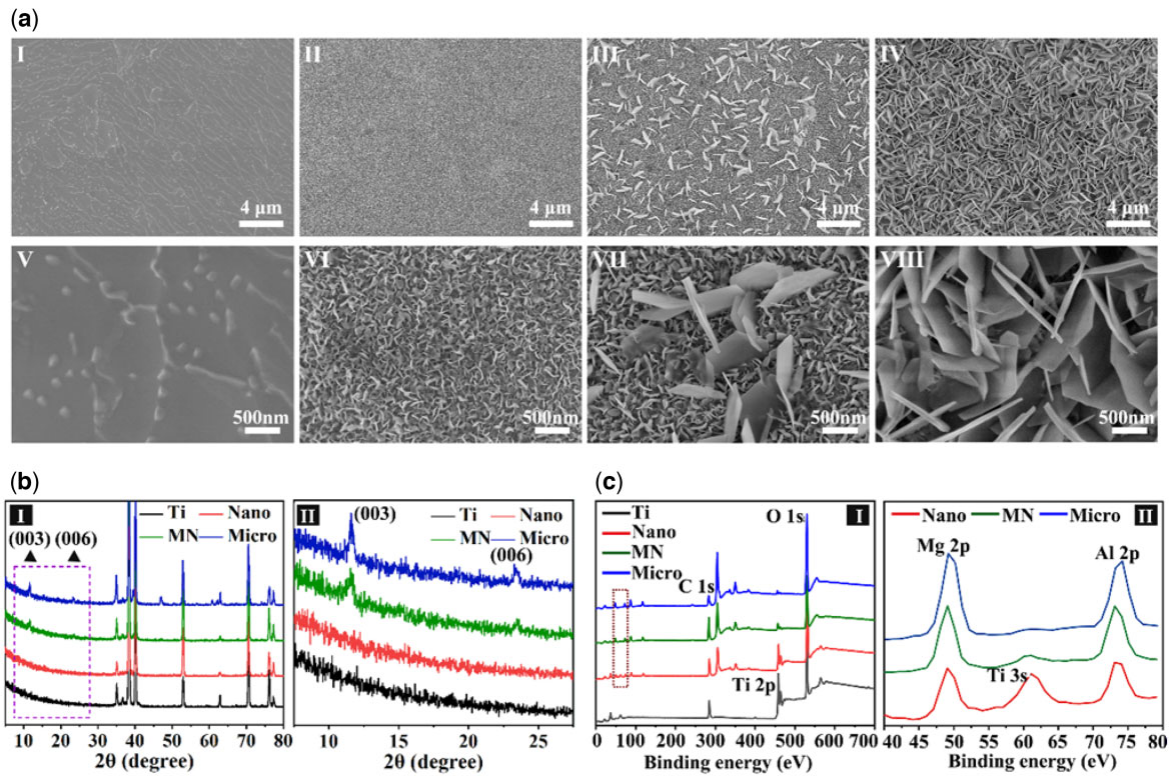
Surface characterizations of various samples. (**a**) SEM images of the surface morphologies of various samples (I, II, III and IV present Ti, Nano, MN and Micro, respectively). (**b**) XRD patterns of samples. (**c**) XPS spectra of samples.

The morphologies of the sample surfaces were tested by SEM and shown in Fig. 1a. After mixed acid treatment, the surface of the Ti sample was flat with and micron-scale gullies. After hydrothermal treatment, the surface of samples exhibited regular sheet structures perpendicular to the substrate with different sizes: nano, micron/nano mixture and micron, which were in accord with the typical sheet array structure of LDHs film. According to the size of sheet structures on samples, the samples were denoted as ‘Nano’, ‘Micro’ and ‘MN’, respectively. The hexagonal side length of sheet structures on Nano and Micro samples were 74.5 ± 2.8 nm and 0.854 ± 0.030 μm, respectively. Those of sheet nanostructures and microstructures on the MN sample were 76.6 ± 5.5 nm and 0.648 ± 0.012 μm, respectively. The sheet nanostructures on the MN sample were continuous and uniform, as were the nanostructures on the Nano sample and the microstructures on the Micro sample, while the microstructures on the MN sample were discontinuous and the distance between these was 1.28 ± 0.192 μm.

XRD patterns of various samples were shown in Fig. 1b. Figure 1b, II was a magnification of the dashed part in Fig. 1b, I. Two diffraction peaks centered at 2θ = 11.6° and 2θ = 23.2°, which corresponded to the characteristic peak of Mg-Al LDH [003] and [006] crystal face, was detected in the XRD patterns of the samples with nano/micro-structures [23]. These diffraction peaks of the Micro and MN sample were obvious, while those of the Nano sample was not obvious due to its thin film thickness and small size of the LDH sheet. Nevertheless, the EDS mapping results (Supplementary Fig. S2) indicating that the surfaces of Nano, MN, and Micro samples were composed of Mg, Al, O and Ti elements, which uniformly distributed on these samples, indicating the same chemical component of these samples. The chemical compositions of all samples were also analyzed by XPS. The XPS full spectra of all samples were shown in Fig. 1c, I and the Mg 2p and Al 2p spectra of modified Ti samples were exhibited in Fig. 1c, II. Only Ti 2p and O 1s peaks can be detected from the Ti sample. The peak strength of Ti 2p on modified Ti samples significantly decreased compared with that of the Ti sample, and the characteristic peaks of Mg 2p and Al 2p appeared on the Nano, Micro and MN surfaces. Ti 3s peak appeared between Mg 2p and Al 2p characteristic peaks on the Nano sample surface because its Mg-Al LDHs film was too thin so that the XPS signal of Ti substrate were detected. The above results confirm that three kinds of Mg-Al LDHs sheet-array films with different sizes (nano, micro and nano/micro mixture) were successfully constructed on the titanium.

### Immunological evaluation

**Figure 2.**
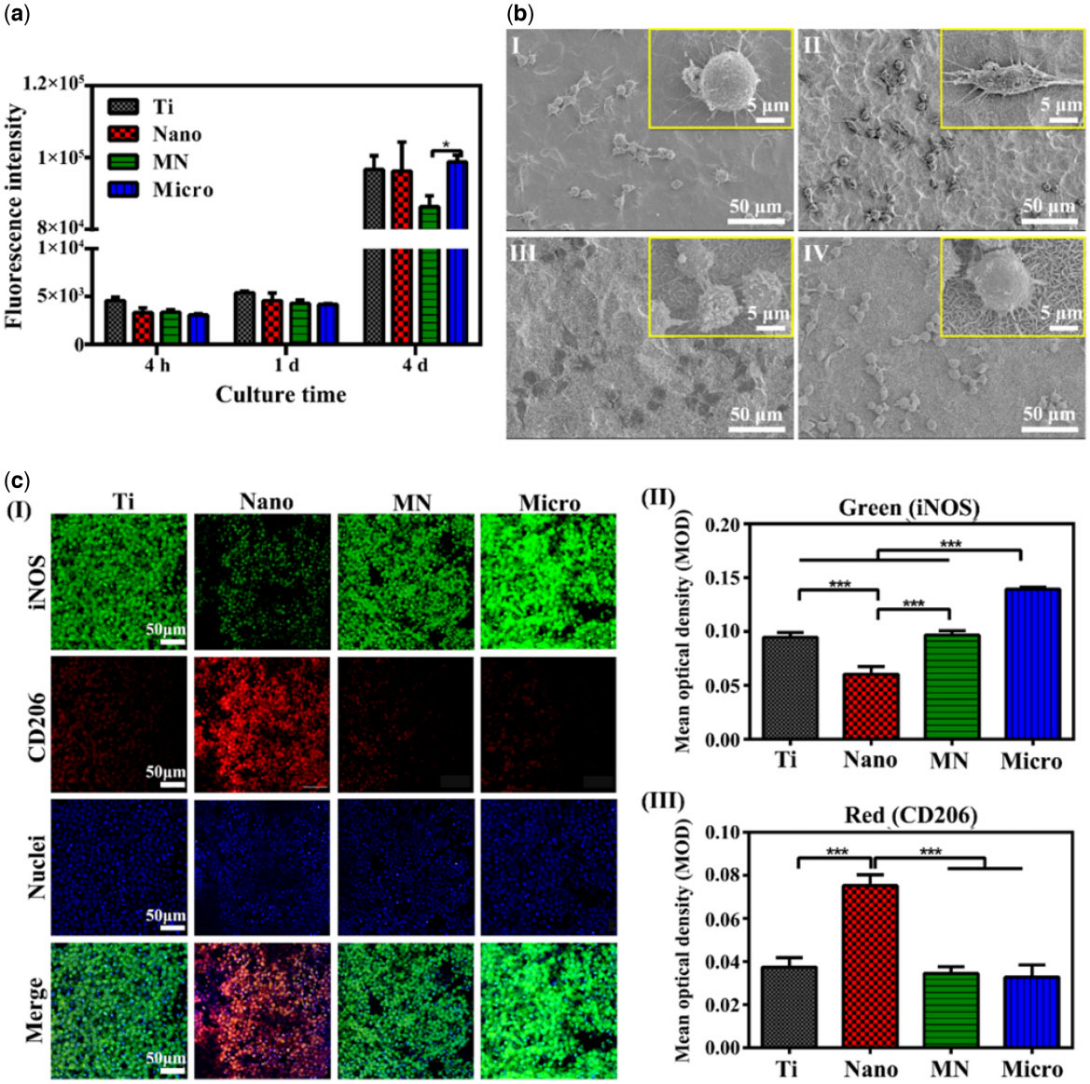
The cytocompatibility, morphologies and polarizations of macrophages on different samples. (**a**) Cell proliferation of macrophages on various samples at 4 h, 1 day and 4 days. Immunofluorescent staining images of macrophages. (**b**) SEM morphologies of macrophages after cultured for 1 day on samples (I, II, III and IV present Ti, Nano, MN and Micro, respectively). (**c**) Immunofluorescent staining images of macrophages on samples cultured for 4 days and the corresponding mean optical density; CD206 was selected as M2 phenotype marker (red), iNOS was selected as M1 phenotype marker (green) and cellular nuclei were stained with 4′,6′-diamidino-2-phenylindole (blue).

The proliferation of macrophages on the sample surface was shown in Fig. 2a. When cultured for 4 h, 1 day and 4 days, the number of macrophages on the Nano, Micro and MN samples were not significantly different from that of the Ti sample, indicating that the modified Ti samples had no obvious cytotoxicity. The SEM morphologies of macrophages cultured for 1 day on the samples were shown in Fig. 2b and exhibited differences on various samples. Macrophages on the Ti surface were round with many slender filopodia. Cells on the MN sample were also round with elongated filopodia, but the number of their filopodia was less than those of cells on the Ti sample. Macrophages on the Micro sample spread well and presented polygonal, their filopodia were many and thin, but shorter compared with the Ti sample. Cells on the Nano sample resembled spindle, and the cell filopodia were short and thick. These results indicated that the effect of LDH structure on the adhesion and spread of macrophages was significantly different. Previous studies have shown that the morphology of macrophages revealed their phenotype and immunological functions [24, 25]. The elongated macrophages showed the characteristics of the M2 phenotype and promoted the secretion of anti-inflammatory cytokines, such as Arg-1 and IL-10 [24].

To intuitively observe the polarization of macrophages, the immunofluorescence staining experiment was carried out. CD206 and iNOS were used to mark M2 and M1 phenotypes, respectively. The polarization of macrophages could be visually observed according to the intensity of red (M2) and green (M1) fluorescence. Images (I) and the mean optical density of macrophages on the samples cultured for 4 days were shown in Fig. 2c. All sample surfaces were covered with cells. The green fluorescence intensity of cells on the Micro sample was the highest among all groups (Fig. 2c, II), indicating that macrophages were more transformed into M1 phenotype and promoted the inflammatory response. That of cells on the Nano sample was the lowest among the four groups, indicating that there were fewer M1 phenotype macrophages. The trend of the green fluorescence intensity of cells on samples showed as follows: Micro > MN ≈ Ti > Nano. On the contrary, the red fluorescence intensity of macrophages on the Nano sample surface was the highest (Fig. 2c, III), indicating that there were a large number of M2 phenotypes, while there was no significant difference in the red fluorescence intensity of macrophages on the other samples. These results indicated that the Micro sample could promote the polarization of M1 macrophages, while the N sample could significantly induce the polarization of M2 macrophages and enhance the anti-inflammatory effect.

**Figure 3.**
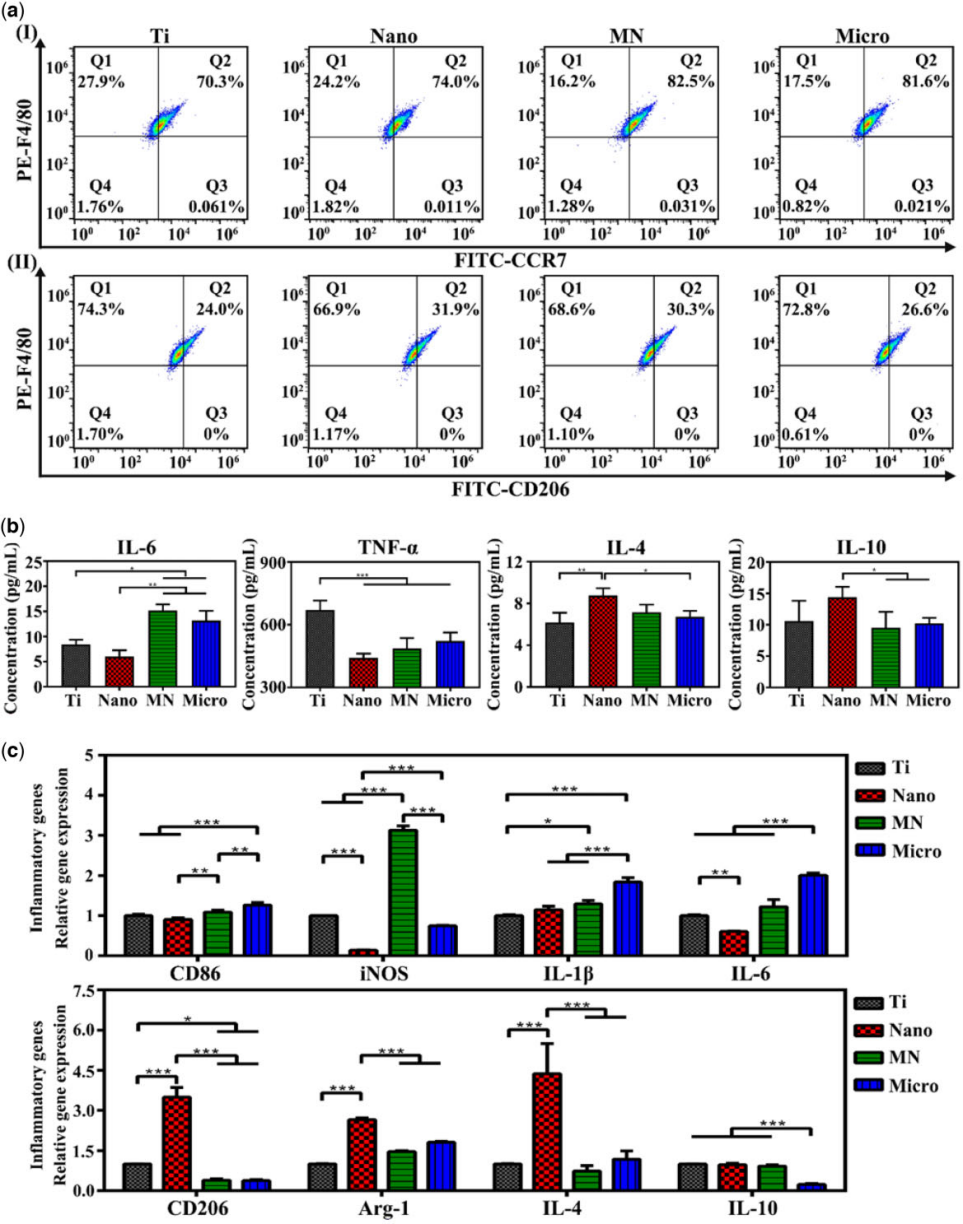
Polarizations and immune responses of macrophages on samples. (**a**) Flow cytometry analyses of cell-surface markers on macrophages (I presents the expressions of F4/80 and CCR7; II presents the expressions of F4/80 and CD206). (**b**) ELISA results of cytokines secreted from macrophages cultured for 4 days on samples. (**c**) Relative mRNA expressions of immune-related genes in macrophages at Day 4 cultured on samples.

Flow cytometry was used to quantitatively determine the polarization of macrophages cultured for 4 days on samples and the results were shown in Fig. 3a and Supplementary Table S3. F4/80 was selected to mark macrophages, and CCR7 and CD206 were chosen as the markers of M1 (Fig. 3a, I) and M2 (Fig. 3a, II) phenotypes, respectively. The proportions of M1 and M2 phenotypes on the Nano, MN and Micro samples were significantly higher than those on Ti samples, indicating that both nanostructures and microstructures can promote the polarization of macrophages. The proportion of M1 macrophages on the samples showed the following trend: MN > Micro > Nano > Ti. While the trend of the proportion of M2 macrophages was as follows: Nano > MN > Micro > Ti. The ratio of M2/M1 macrophages on the samples exhibited the following trend: Nano > MN > Ti > Micro. The results showed that the macrophages on the Nano sample were prone to the polarization of the M2 phenotype and would have the most obvious anti-inflammatory effect among the four groups.

Cytokines in cell culture medium released by macrophages on samples were detected by ELISA, as shown in Fig. 3b. After cultured for 4 days, macrophages on the Nano sample secreted the most anti-inflammatory factors (IL-4 and IL-10) and the least pro-inflammatory factors (IL-6 and TNF-α) among the four groups. The amount of inflammatory factor TNF-α released by macrophages on the Nano, MN and Micro samples was significantly lower than that of Ti samples. The amount of IL-6 released from macrophages on the Micro and MN samples was higher than that of Ti and Nano samples. According to the results, it could be concluded that the inflammatory response of macrophages on the surface of the Nano sample is the weakest.

The expression of immune-related genes in macrophages was detected by RT-PCR and the results were shown in Fig. 3c. The expression of CD206 (M2 phenotype marker) in cells on the Nano sample was the highest, while that of MN and Micro sample was lower than that of Ti sample, and there was no significant difference between the MN and Micro samples. The expression of the M1 macrophage marker CD86 gene on the Micro sample was the highest, while that of the Nano sample was the lowest. Moreover, the expression levels of anti-inflammatory genes Arg-1 and IL-4 in macrophages on the Nano sample were the highest, but the expression of inflammatory genes iNOS and IL-6 were the lowest. The expression of inflammatory genes IL-6 and IL-1β in macrophages on the Micro sample were the highest among the four groups, while the expression levels of anti-inflammatory genes IL-10 in cells on the Micro sample were the lowest. The trend of anti-inflammatory gene expression in macrophages on the samples was as follows: Nano > Ti > MN > Micro, and the trend of inflammatory gene expression was as follows: Micro > MN ≈ Ti > Nano.

### Induction of osteogenic differentiation by macrophages

To better simulate the situation of material implantation in the human body, mBMSCs were directly seeded on the samples, then cultured in the macrophage-conditional medium to study the synergistic effect of samples and macrophages on the osteogenic differentiation of stem cells.

**Figure 4.**
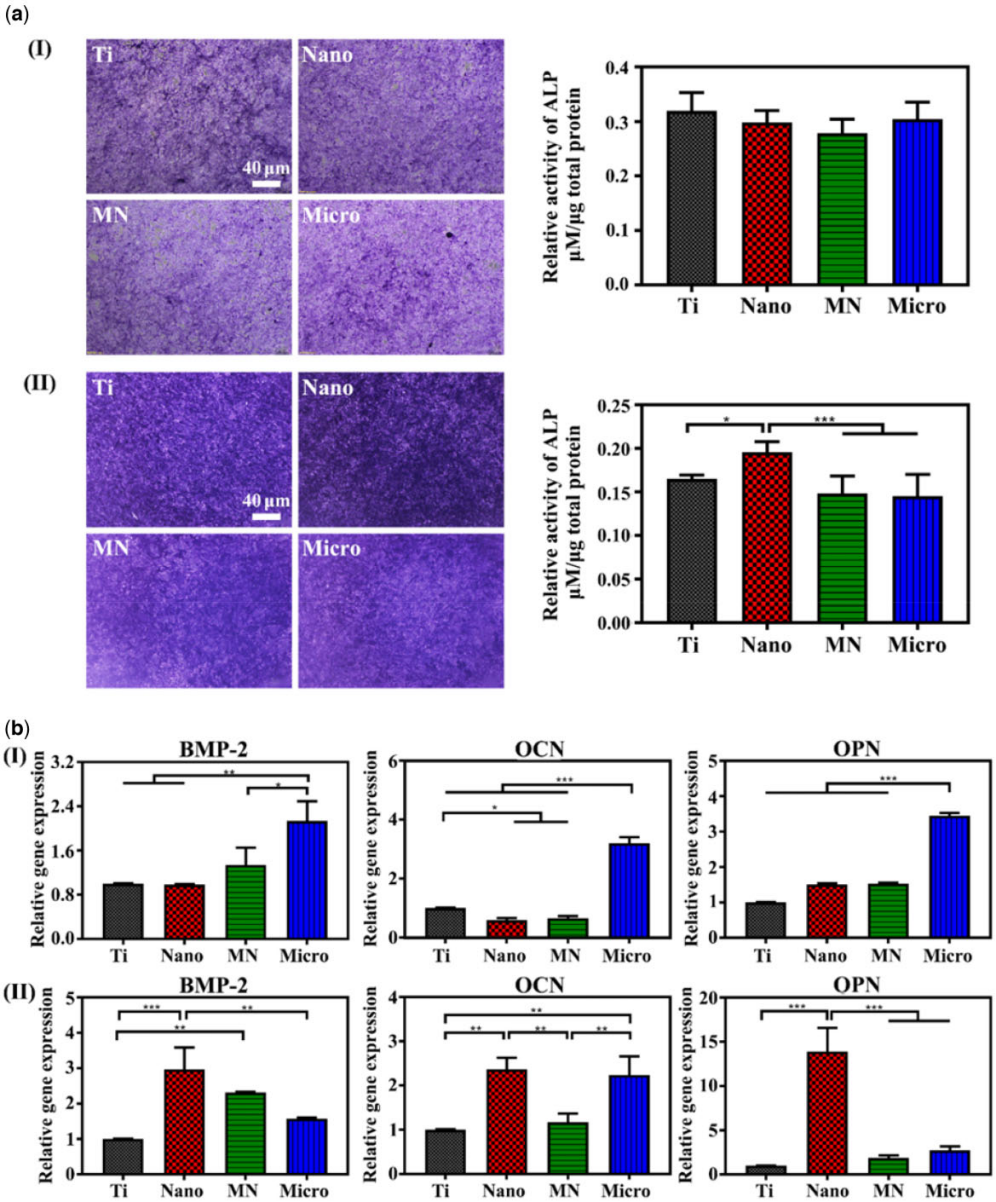
Osteogenic activity of mBMSCs on samples. (**a**) ALP positive areas of mBMSCs cultured on various samples for 10 days and the corresponding colorimetrically qualitative results (I presents the single-cultured mBMSCs; II presents the indirect co-cultured BMSCs). (**b**) Relative mRNA expression levels of the osteogenic genes in mBMSCs at Day 10 on sample surfaces (I presents the single-cultured mBMSCs; II presents the indirect co-cultured BMSCs).

ALP staining and ALP activity experiments were conducted to investigate the effect of macrophages on BMSCs’ osteogenic differentiation. The ALP activities of mBMSCs cultured on the samples were shown in Fig. 4a, I. There was no obvious difference in the ALP activity of mBMSCs on all samples. Figure 4a, II showed the result of ALP activity of BMSCs cultured in the conditional medium on the samples. The ALP activity of mBMSCs on the Nano sample was the highest among the four groups, and there was no significant difference in the ALP activity among the other three groups. The results indicate that these nano/micro-sheet array structures had little effect on the osteogenic differentiation of mBMSCs. However, the secretions of M1 macrophages (e.g. IL-1β, TNF-α) can inhibit osteogenic differentiation, especially TNF-α cytokines, the amounts of TNF-α in the macrophage culture medium of all groups were significantly higher than those of other cytokines (Fig. 3b). The osteogenic differentiation of the co-cultured mBMSCs in each group was decreased with the inhibition of TNF-α, manifested by the decreased activity of ALP. Moreover, because immune responses of macrophages on different samples vary, the Nano sample with the highest proportion of M2/M1 macrophages can better promote the ALP activity of mBMSCs, while the Micro sample with the lowest ratio of M2/M1 macrophages caused the lowest ALP activity of mBMSCs, indicating that macrophages on the Nano sample could promote the osteogenic differentiation of mBMSCs.

Figure 4b, I showed the expression of osteogenic genes in mBMSCs cultured on the sample surface for 10 days. The expression of BMP-2, OPN and OCN genes in mBMSCs on the Micro sample was the highest, and there was no significant difference between the Ti, Nano and MN samples. However, when mBMSCs were cultured in the macrophage-conditioned medium on samples for 10 days, the expression of osteogenic genes in cells was different (Fig. 4b, II). The expression of BMP-2, OPN and OCN genes in mBMSCs on the Nano sample was the highest among the four groups. Especially, the expression of BMP-2 and OPN in mBMSCs on the Nano sample was significantly higher than those of the Micro sample. In conclusion, the microstructures on the Micro sample could upregulate the osteogenic gene expression in mBMSCs and promote mBMSCs osteogenic differentiation compared with nanostructures and the Ti sample. While the macrophages on the Nano samples can significantly upregulate the osteogenic gene expression in mBMSCs. The induction of osteogenic differentiation by macrophages was superior to the osteogenic effect of the microstructures. These results are due to that the macrophages on the Nano sample are mainly M2 phenotype, and their secretions can upregulate the expression of the osteogenic gene in mBMSCs and further promote the differentiation of BMSCs into osteoblasts.

## Discussion

Cells are exposed to a complex environment consisting of various structural features in the human body, which affect cell biological behaviors to varying degrees, including adhesion, proliferation and differentiation. It is believed that the research of appropriate micro or nano structures mimicking the structure of natural bone is extremely important for intraosseous implants to achieve osseointegration. Recent studies have revealed that surface morphology dictated important aspects of cell osteogenic differentiation [1, 26, 27]. However, the role of immune response in the process of bone-material integration is always neglected. In this work, a series of micro/nano-sheet array LDHs films were directly grown on the titanium surface by hydrothermal treatment to investigate the effect of the size of sheet array structure on the immune response and consequent osteogenic differentiation. The Mg-Al LDH structures observed by SEM were in different dimensions: nano, micro and micron/nano mixture, respectively. XRD and XPS results confirmed that the films on the modified Ti surfaces were in the same composition, Mg-Al LDHs.

Topography-induced changes in cell morphology could be directly observed in the early cell adhesion. In this work, although the proliferation of macrophages on the modified samples was not obviously inhibited compared with that on the Ti sample (Fig. 2a), the cell adhesion and spreading behaviors were observed by SEM on the samples with various sheet structures were significantly different (Fig. 2b). Macrophages on the Nano sample resembled spindle, and the cell filopodia were short and thick, while they on other samples were round with thin filopodia. Hence, focal adhesions were further studied because they are the important transducers of mechanical cues such as the topography from the previous studies. Integrin, consisting of α and β subunits, is an important class of receptors involved in cell adhesion and spread and it physically connects to the actin cytoskeleton through a series of cytosolic adaptor proteins [28, 29]. Cellular ‘inside-out’ signaling influences the affinity of integrin and ECM proteins to control adhesion strength and enable sufficiently strong interactions between them, and further to transmit the forces required for cell spread and ECM remodeling [30, 31]. Also, integrin can act as traditional signaling receptors in transmitting information into cells [32]. In general, integrin plays central a role in the biology of metazoan cells by affecting cell adhesion to ECM and cell survival, polarity, cytoskeletal structure and apoptosis.

**Figure 5.**
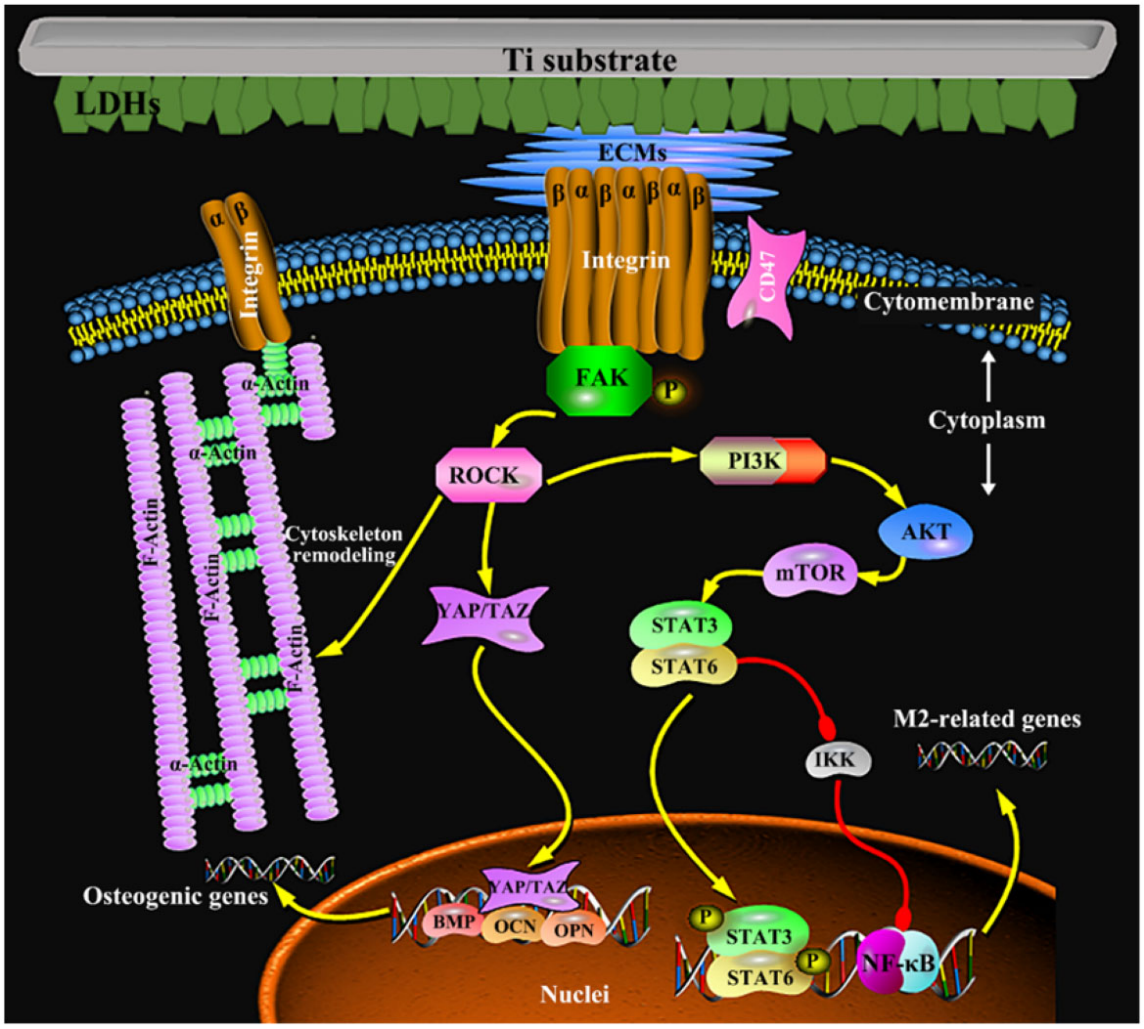
The integrin-related signaling pathway mediates the immune response and the osteogenesis between macrophages and mBMSCs on sample surfaces.

Therefore, the cell adhesion on the surface of biomaterials was investigated in this work. As shown in Fig. 5, the gene expressions of adhesion-associated genes (integrin β2 and FAK) in macrophages on the Nano sample were the highest, while those in cells on the MN sample were the lowest among the four groups. The possible reason may be that the average integrin interspacing of focal adhesions and the adhesion-related particles are in nano dimension [33], while the hexagonal side length of sheet array structures on the Nano sample is also in nano size. The uniformly distributed nano-scale features on the surface of the Nano sample substantially match the desirable interspacing of integrins to form focal adhesions, therefore improving cell adhesion. However, the hexagonal side lengths of LDHs in micron size on the MN and Micro samples are much bigger and they do not significantly facilitate cell adhesion, especially the MN sample with micron lamellar spacing.

**Figure 6.**
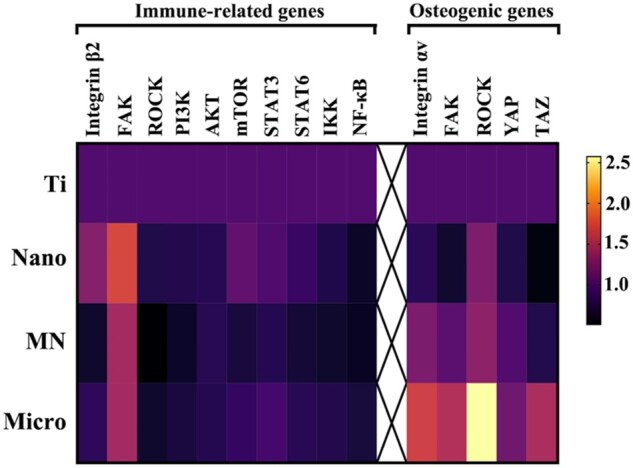
PCR results of integrin-related gene levels in macrophages and mBMSCs at Day 4 cultured on samples.

Moreover, the downstream signaling of integrin in regulating the M2 polarization of macrophages was further investigated in this study (Fig. 5), showing that the most likely signal transduction pathway to participate in adhesion-induced polarization of macrophages may be the PI3K-AKT-mTOR signal pathways (Fig. 6) [34]. The outcomes showed that among the modified Ti samples, the PI3K-AKT-mTOR signaling pathway was activated in macrophages on the Nano sample, combing with high gene expressions of integrin β2 and FAK. While the gene expression of the PI3K-AKT-mTOR signaling pathway in macrophages on the MN sample was the lowest among the three modified samples with the low gene expressions of integrin β2 and FAK. Moreover, the gene expressions of downstream signaling of mTOR in macrophages were also studied. The gene expressions of STAT3 and STAT6, which mediate the anti-inflammation [35, 36], in cells on the Nano sample were the highest and those of STAT3 and STAT6 in cells on the MN sample were the lowest among the modified Ti samples, but there was no significant difference in the gene expressions of IKK and NF-κB in cells on all modified samples. The results of these gene expressions were consistent with the phenotype of macrophages on these LDH films, that is, macrophages on the Nano sample were more likely to polarize into the M2 phenotype with the best anti-inflammatory effect.

The surface morphology of LDH film has been considered as the vital factor to manipulate the cytoskeleton and focal adhesion status of mBMSCs and further regulate the osteogenic differentiation (Fig. 5). As the diameter of mBMSCs is about 10 times bigger than that of macrophages, the recognition and adhesion of mBMSCs on LDH film are different from that of macrophages and more susceptible to the large size of LDH sheet array structures. In the previous work, it was found that stem cells (osteoblasts) that spread on the surface of micro/nano structures are more than 40 μm in diameter, and their pseudopodia can be tens of microns in size [22]. Focal adhesions (FAs) are linked to F-actin and the myosin II, the formation of mature FAs is important for the regulation of cell adhesion, mechanical sensing and the cell growth and differentiation [29]. FAs formation is closely related to the spacing of the integrin ligand (integrin clustering), and a threshold of 60–70 nm is found to be necessary for FAs formation and tension development [37]. Clustering of integrins may only be required to occur at a local scale (4–5 integrins) to form FAs and the area of mature FAs is usually several μm^2^ [38]. Compared with LDH nanosheets, micro-sheet arrays are more conducive to FAs formation in stem cells; alternatively, the micron spacing will generate greater tension, and elongated FAs are formed and connect to better structured and more mature actin fibers. Furthermore, the gene expressions of integrin αv and FAK in mBMSCs on the Micro sample were the highest, while those of integrin αv and FAK in mBMSCs on the Nano sample were the lowest among the four groups, which are significantly different from those of integrin β2 and FAK in macrophages on samples. Moreover, the PCR result showed that the gene expressions of ROCK, YAP and TAZ in mBMSCs on the Micro samples exhibited the highest, which resulted in cytoskeletal rearrangement and the increased expression of osteogenic genes [7, 39]. Furthermore, when mBMSCs were cultured in the conditional medium of macrophages, the osteogenic differentiation of them on the modified Ti samples changed obviously. The gene expressions of osteogenic differentiation and ALP activity in the co-cultured mBMSCs on the Nano sample became the highest rather than the Micro sample among three modified groups. This may be attributed to the high proportion of M2 macrophages on the Nano samples, which can promote the osteogenic differentiation of mBMSCs by secreting osteogenic factors, and this promoting effect is significantly better than the regulation effect of material surface morphology. The relevant mechanisms require further research and exploration.

Above all, this study suggested that the regulation mechanisms of the surface topography with different sizes induced macrophages polarization was closely related to the cell adhesion behavior on the sample surface, which may be relevant to the PI3K-AKT-mTOR signaling pathway. Moreover, the nano-sheet array structured titanium surface with the highest proportion of M2 macrophages promoted the osteogenic differentiation of mBMSCs, which provides an idea for surface design of orthopedic biomaterials that is inducing appropriate M2 macrophages *via* biomaterial surface engineering for further osteogenesis.

## Conclusion

Three various sizes of Mg-Al LDH sheet array structures, including nano, micro and nano/micro mixture, were directly grown on the surface of biomedical titanium by hydrothermal treatment. The surface with nano-sheet array structures significantly promoted the polarization of M2 macrophages by activating the PI3K-AKT-mTOR signaling pathway with high gene expressions of integrin β2 and FAK. While the surface with micro-sheet array structures enhanced osteogenic differentiation of mBMSCs *via* ROCK-YAP/TAZ-mediated mechanotransduction. Moreover, the indirect co-culture model assay exhibited that the nano-sheet array structures promoted the osteogenic differentiation of mBMSCs with a high proportion of M2 macrophages through a shared medium. This study gave further information concerning integrin-induced focal adhesions in cells of different sheet array structures and their role in macrophages polarization and osteogenic differentiation of mBMSCs, which might clarify the targeted modulation of integrin-mediated mechanotransduction by building the optimum geometry for advanced biomaterials.

## Supplementary data

Supplementary data are available at *REGBIO* online.

## CRediT authorship contribution statement

X.Z.: Experiments; Data Curation; Writing-Review & Editing. L.C.: Biological experiment design and analysis; Writing-original draft; Writing-Review & Editing. J.T.: Conceptualization; Methodology; Material design and optimization; Writing-Review & Editing; Supervision. J.M.: Writing-Review & Editing. X.L.: Writing-Review & Editing. T.Y.: Methodology; Writing-Review & Editing; Supervision. Z.D.: Methodology; Writing-Review & Editing; Supervision.

## Funding

This work was financially supported by the National Natural Science Foundation of China (51831011 and 31870944), Medical discipline Construction Project of Pudong Health Committee of Shanghai (PWYts2021-05), and Postdoctoral Science Foundation of China (2021 M693260).

*Conflicts of interest statement*. The authors have no competing interests to declare.

## Supplementary Material

rbac075_Supplementary_Data
